# Poor mental health and sexual risk behaviours in Uganda: A cross-sectional population-based study

**DOI:** 10.1186/1471-2458-11-125

**Published:** 2011-02-21

**Authors:** Patric Lundberg, Godfrey Rukundo, Schola Ashaba, Anna Thorson, Peter Allebeck, Per-Olof Östergren, Elizabeth Cantor-Graae

**Affiliations:** 1 Division of Social Medicine, Department of Public Health Sciences, Karolinska Institute, Stockholm, Sweden; 2 Division of Social Medicine and Global Health, Department of Clinical Sciences, Lund University, Malmö, Sweden; 3 Department of Psychiatry, Mbarara University of Science and Technology, Mbarara, Uganda; 4 Division of Global Health (IHCAR), Department of Public Health Sciences, Karolinska Institute, Stockholm, Sweden

## Abstract

**Background:**

Poor mental health predicts sexual risk behaviours in high-income countries, but little is known about this association in low-income settings in sub-Saharan Africa where HIV is prevalent. This study investigated whether depression, psychological distress and alcohol use are associated with sexual risk behaviours in young Ugandan adults.

**Method:**

Household sampling was performed in two Ugandan districts, with 646 men and women aged 18-30 years recruited. Hopkins Symptoms Checklist-25 was used to assess the presence of depression and psychological distress. Alcohol use was assessed using a question about self-reported heavy-episodic drinking. Information on sexual risk behaviour was obtained concerning number of lifetime sexual partners, ongoing concurrent sexual relationships and condom use.

**Results:**

Depression was associated with a greater number of lifetime partners and with having concurrent partners among women. Psychological distress was associated with a greater number of lifetime partners in both men and women and was marginally associated (p = 0.05) with having concurrent partners among women. Psychological distress was associated with inconsistent condom use among men. Alcohol use was associated with a greater number of lifetime partners and with having concurrent partners in both men and women, with particularly strong associations for both outcome measures found among women.

**Conclusion:**

Poor mental health is associated with sexual risk behaviours in a low-income sub-Saharan African setting. HIV preventive interventions should consider including mental health and alcohol use reduction components into their intervention packages, in settings where depression, psychological distress and alcohol use are common.

## Background

Is poor mental health associated with sexual risk behaviours in sub-Saharan Africa? This question has not been conclusively answered despite its relevance for HIV prevention[1,2].

Poor mental health is a major cause of disability in low-income countries, with depression constituting the heaviest disease burden[3]. The HIV epidemic may contribute to increased depression rates in countries having high HIV prevalence: HIV may lead to depression both in persons who live with HIV[4,5] and in those who are indirectly affected[6-8]. An association between depression and sexual risk behaviours is thus particularly relevant in low-income countries with high HIV prevalence.

In high-income countries, poor mental health has been closely linked to risky sexual behaviours[9]. For instance, longitudinal studies from the United States suggest that depressive symptoms contribute to sexual risk behaviours (multiple sexual partners, unprotected sex) in the general population, with potential mechanisms including maladaptive coping, low self-efficacy, and self-destructiveness[10-12]. However, the relationship between depressive symptoms and sexual risk behaviours may be non-linear: persons having moderate, but not severe, depressive symptoms may engage in the most sexual risk behaviours[13].

However, conclusions derived from findings in high-income countries may not be applicable to low-income settings. Sexual behaviours vary widely between and within countries, and are determined both by context and individual factors[14]. In many sub-Saharan African low-income countries, contextual factors such as poverty/wealth, mobility, and gender inequality heavily influence sexual behaviours[14-18]. In contrast, in high-income countries, personal choice may have a greater influence.

Nevertheless, to our knowledge only three studies have previously investigated the association between poor mental health and sexual risk behaviours in the general population in sub-Saharan African countries: In South Africa, depressive symptoms predicted transactional sex and intimate partner physical and sexual violence in women, and inconsistent condom use in men [19], while cross-sectional associations with sexual risk behaviours have also been found [19,20]. In Botswana, cross-sectional associations were found between depressive symptoms and having multiple partners among women, and with paying for sex among men [21]. In addition, South Africa and Botswana are both middle-income countries, and to our knowledge, no population-based study has investigated the association between poor mental health and sexual risk behaviours in a *low-income *sub-Saharan African setting.

Thus, we investigated the association of (1) depression, (2) psychological distress, i.e. depressive and anxiety symptoms, and (3) alcohol use, with sexual risk behaviours in young Ugandans in the general population. Uganda is a low-income country with a generalised HIV epidemic (prevalence in 2005: 6.4%[22]). Moreover, population-based surveys suggest that mental health problems are very common in the country (depression prevalence range: 10-50%)[23-26], and mental health services are sparse with 0.8 psychiatrists per one million population[27].

## Method

### Participants

The study took place in Kampala (mainly Baganda ethnic group) and Mbarara district (mainly Banyankole ethnic group). The Baganda and Banyankole are culturally and linguistically related.

We performed a cross-sectional population-based study in nine purposively selected study areas representing varying degrees of urbanicity in Uganda: 3 divisions in Kampala city (urban), 3 divisions in Mbarara town (semi-urban), and 3 sub-counties in Mbarara district (rural). Persons aged 18-30 years, residing in these areas and not reporting or displaying overt signs of severe mental or physical illness, and not under the influence of alcohol or drugs at the time of contact, were eligible. This age group was selected because it was deemed to represent the segment of the adult population being most sexually active.

In each study area, seven villages (in rural areas) or neighbourhoods (in semi-urban/urban areas) were purposively selected, in order to reflect the range of levels of economic development of villages/neighbourhoods in the study area. From a central location in each selected village/neighbourhood, a walk was performed in a random direction, towards the end of the village/neighbourhood. The random direction was obtained spinning a pen and observing in what direction the pen pointed after the spinning had stopped. Every third household encountered during the walk was visited, until ten interviews had been conducted, thus yielding an approximate sample size of 9 × 7 × 10 = 630 participants in total. All eligible persons at home were interviewed. If no eligible person was at home the next household was visited, and then, every third household. Follow-up visits were not performed. In four of the 21 selected neighbourhoods in Kampala, household sampling proved not to be feasible using our random walk procedure, given a low density of accessible households and a predominance of shops and businesses in the neighbourhood. In these neighbourhoods a pragmatic approach was adopted: Instead of households, shops and businesses were sampled using the same systematic sampling strategy as that used for households, with participants systematically recruited inside the selected establishments.

Among the persons who were approached and invited for participation, refusals were rare (estimated at <5%). Data was collected between September 2004 and June 2005 by seven Ugandan field workers (university students). Initial training focussed on interviewing techniques and ethical considerations specific to interviews about mental problems and sexual behaviours. Interviewers and respondents were not sex-matched. Continuous supervision was provided throughout the field-work by two of the authors (G.R. and S.A.). Study languages were English, Luganda and Runyankole, depending on the proficiency of the respondent. Informed consent was sought and the risks, benefits and right to decline or withdraw were carefully explained. Although potential participants were sometimes approached in the vicinity of other persons, after informed consent had been obtained, the research assistant and the participant withdrew to a more secluded location where privacy was ensured.

All study procedures were approved by the Institutional Ethical Review Committee of Mbarara University.

### Measures

*Socio-demographic background factors *were: age (18-24 yrs vs. 25-30 yrs), level of education (up to primary school vs. more than primary school), relationship status (single/widowed/separated vs. married/cohabiting), and place of residence (urban vs. semi-urban/rural with urban referring to Kampala district).

*Depression *was assessed using the depression sub-section of the Hopkins Symptoms Checklist (HSCL-25)[28]. This instrument was developed for cross-cultural use and consists of 25 questions assessing symptoms of anxiety (10 items) and depression (15 items) during the past week, with the response to each item graded from 1 = "not at all" to 4 = "extremely". The depression sub-section of the questionnaire (15 items) has been validated among the Baganda as a screening tool for probable depression, with a cut-off point established based on comparison with structured clinical interviews[26,29]. Thus, we categorized participants having a depression sub-scale total score of 31 or above as having probable depression[26], in order to be able to assess the association of clinical depression with sexual risk behaviours. The Chronbach's alpha for the depression sub-scale was 0.84, i.e. similar to that previously reported in Uganda[26].

*Psychological distress *was assessed by calculating a total score for the entire HSCL-25 instrument including both depression and anxiety items[28] as has been done previously[30,31]. Depression and psychological distress only partly overlap. Thus, many persons in the general population have psychological distress but do not fulfil diagnostic criteria for clinical depression. Nevertheless, such persons might potentially have increased sexual risk behaviours. Therefore, we used the HSCL-25 as a continuous measure of psychological distress and investigated whether this measure was associated with sexual risk behaviours. The continuous psychological distress variable was categorised into gender-specific quartiles instead of using e.g. a dichotomous measure, in order to capture sub-clinical psychological distress, and given that a non-linear relationship between psychological distress and sexual risk behaviour was deemed possible[13]. Gender-specific quartiles were used in order to maximise statistical power for within-gender analyses of the association between psychological distress and sexual behaviours. The 1^st ^quartile (i.e. least psychological distress) was used as the reference category to which the other HSCL-25 quartiles were compared. The Chronbach's alpha for the entire instrument was 0.92, indicating excellent internal consistency, and suggesting that all HSCL-25 items could indeed be used to measure a common underlying construct.

*Alcohol use *referred to heavy episodic drinking, a behaviour associated with sexual risk taking[21,32,33], and was operationalised as the number of times 'drunk on alcohol' per week (0 vs. 1 or more). Self-reports of heavy episodic drinking have previously been used in sub-Saharan African contexts[34] and may provide a viable alternative in settings where estimating the number of 'standard drinks' is difficult: In rural Uganda, a significant proportion of the alcohol consumed is locally produced, has variable alcohol content and is consumed from plastic bags, cups or a common pot.

*Sexual behaviours *were assessed using three measures:

(1) 'Number of lifetime sexual partners'

Sexual partner was defined as a person with whom one has ever had sexual intercourse. The number of lifetime sexual partners was categorized as 0-3 vs. 4 or above, approximately corresponding to the 75^th ^percentile in the current sample.

(2) 'Number of current sexual partners'

Current sexual partner was defined as a partner with whom one currently has sexual intercourse regularly. The number of current sexual partners was categorized as 0-1 vs. 2 or more. This measure thus targeted ongoing regular sexual relationships as has been done previously[35], in order to estimate the point prevalence of concurrency[36]. Two or more current sexual partners are hereafter referred to as concurrent sexual partners.

(3) 'Frequency of condom use when having sex'

This question assessed the conditional likelihood that the person uses a condom in case he or she has sexual intercourse, and therefore used a relative measure of condom use (always, sometimes, never) and did not have a specific reference time period. Conceptualizing condom use as a habit, and using relative instead of count measures may increase sensitivity when investigating the psychological correlates of unprotected sex[37]. Condom use was categorised as always (consistent) vs. sometimes/never (inconsistent) condom use.

The study instrument was translated from English into Runyankole and Luganda and independently back-translated. The two versions were compared and necessary adjustments made (P.L., G.R., S.A. and translators). Care was taken in order not to introduce culturally alien or offensive notions or expressions, while ensuring that the intended construct was indeed being measured. The questions about sexual behaviours were asked at the end of the interview for increased acceptability. After back-translation and modification, the questionnaire was pre-tested on persons not participating in the study, with results indicating that the questions were acceptable and comprehensible.

### Analyses

All analyses were *a priori *stratified by gender. Missing values were excluded from analyses and all percentages are percentages of valid answers. For analyses pertaining to condom use, participants who reported never having had sex were excluded.

Sexual risk behaviours were treated as outcome measures. Depression, psychological distress, and alcohol use were examined as possible predictors. However, depression, psychological distress, and alcohol use may all be inter-related. For instance, alcohol use may be both a consequence and a cause of psychological distress and depression. Thus, each of these measures was examined separately in relationship to sexual risk behaviour. Socio-demographic background factors were included into all models in order to adjust for confounding.

Moreover, in explorative analyses we investigated whether the inter-relatedness between depression, psychological distress and alcohol use influenced their respective associations with the outcome. Thus, we examined whether the associations of depression and psychological distress with sexual risk behaviours were independent of alcohol use, and conversely, whether the association of alcohol use with sexual risk behaviours was independent of depression and psychological distress, respectively. The results of these explorative analyses are presented in the text, but not in the tables.

We used a modified cluster sampling method, with the clusters being villages/neighbourhoods, as contrasted to a genuinely random sampling method. Thus, given that some statistical efficiency may be lost in case responses within clusters correlate, we investigated whether adjustment for such intra-cluster correlation substantially influenced the results obtained, using the Stata *svy *procedure. However, confidence intervals remained virtually unchanged after adjustment and the original confidence intervals are presented in this manuscript. Significance level was set at *p *< 0.05.

## Results

88% of the men and 82% of the women reported ever having had sex. 37% (n = 123) of the men and 23% (n = 70) of the women reported four or more lifetime sexual partners. 15% (n = 49) of the men and 4.5% (n = 14) of the women reported having concurrent sexual partners. 73% (n = 204) of the men and 77% (n = 193) of the women who had ever had sex reported inconsistent condom use.

The prevalence of probable depression was 12.0% among men and 17.9% among women. Heavy episodic drinking at least once per week was reported by 35.4% (n = 118) of the men and 13.3% (n = 41) of the women. The distribution of socio-demographic, mental health and sexual risk behaviour variables are presented in Table 1.

**Table 1:** Sample characteristics

Characteristic	Men, n = 334 n (%)	Women, n = 312 n (%)
**Age**		
18-24 yrs	161 (48.2)	176 (56.4)
25-30 yrs	173 (51.8)	136 (43.6)

**Education**		
<=7 yrs	110 (33.1)	97 (31.3)
>7 yrs	222 (66.9)	213 (68.7)

**Place of residence**		
Urban	115 (34.4)	102 (32.7)
Semiurban/rural	219 (65.6)	210 (67.3)

**Relationship status**		
Single/widowed/separated	178 (53.6)	179 (57.6)
Married/cohabiting	154 (46.4)	132 (42.4)

**Depression**		
No	294 (88.0)	256 (82.1)
Yes	40 (12.0)	56 (17.9)

**Psychological distress^1^**		
Lower quartile	1.16	1.16
Median	1.28	1.36
Upper quartile	1.64	1.80

**Alcohol use^2^**		
Less than once per week	215 (64.6)	268 (86.7)
At least once per week	118 (35.4)	41 (13.3)

**Lifetime sexual partners**		
0	41 (12.4)	56 (18.0)
1	69 (20.9)	89 (28.6)
2	56 (17.0)	60 (19.3)
3	41 (12.4)	36 (11.6)
4 or above	123 (37.3)	70 (22.5)

**Current sexual partners**		
0	98 (29.8)	93 (30.0)
1	182 (55.3)	203 (65.5)
2	42 (12.8)	12 (3.9)
3	4 (1.2)	2 (0.6)
4	3 (0.9)	0 (0)

**Condom use^3 ^(n = 544)**		
Always	77 (27.4)	57 (22.8)
Sometimes	110 (39.1)	109 (43.6)
Never	94 (33.5)	84 (33.6)

^1 ^Values represent HSCL-25 quartiles cut-off scores.

^2 ^Alcohol use refers to self-reported heavy episodic drinking.

^3 ^Condom use was assessed only among those participants who had ever had sex.

### Depression and sexual risk behaviours

Depression was independently associated with a greater number of lifetime sexual partners among women, but not among men, after simultaneous adjustment for all socio-demographic background variables, see Table 2. Similarly, depression was independently associated with having concurrent sexual partners among women, but not among men. Associations between depression and inconsistent condom use did not reach statistical significance in either men or women.

**Table 2:** Association of depression with sexual risk behaviours in 334 men and 312 women aged 18-30 years in the general population in Uganda

	Lifetime partners			Current regular partners			Inconsistent condom use^2^		
	
Depression	Proportion with 4 or more lifetime partners (%)	Crude OR (95% CI)	Adjusted^1 ^ OR (95% CI)	Proportion with more than 1 current partner (%)	Crude OR (95% CI)	Adjusted^1 ^ OR (95% CI)	Proportion with inconsistent condom use (%)	Crude OR (95% CI)	Adjusted^1 ^ OR (95% CI)
**Men**									
No depression	104/290 (35.9)	1	1	41/290 (14.1)	1	1	171/243 (70.4)	1	1
Depression	19/40 (47.5)	1.62 (0.83-3.15)	1.63 (0.78-3.42)	8/39 (20.5)	1.57 (0.67-3.65)	1.52 (0.60-3.86)	33/38 (86.8)	2.78 (1.04-7.41)	2.71 (0.72-10.20)

**Women**									
No depression	47/255 (18.4)	1	1	8/254 (3.1)	1	1	150/199 (75.4)	1	1
Depression	23/56 (41.1)	3.08 (1.66-5.73)	2.74 (1.41-5.34)	6/56 (10.7)	3.69 (1.23-11.10)	3.92 (1.25-12.27)	43/51 (84.3)	1.76 (0.77-3.99)	2.12 (0.79-5.68)

^1 ^Adjustment for age, education, marital status and place of residence.

^2 ^Analyses pertain only to participants who had ever had sex.

When the above results were adjusted for alcohol use in explorative analyses, the association between depression and concurrent sexual partners in women was somewhat attenuated (OR 3.03; 95% CI 0.90-10.24), indicating that alcohol use mediated or confounded some part of that association. However, associations of depression with number of lifetime sexual partners and with condom use, respectively, were independent of alcohol use in both men and women.

### Psychological distress and sexual risk behaviours

Psychological distress was independently associated with a greater number of lifetime sexual partners in both men and women, after simultaneous adjustment for socio-demographic background variables, see Table 3. The association between psychological distress and having concurrent sexual partners did not reach statistical significance in either men or women, although in women the association approached significance (p = 0.05). Psychological distress was associated with inconsistent condom use in men, while in women results did not reach statistical significance.

**Table 3:** Association of psychological distress with sexual risk behaviours in 334 men and 312 women aged 18-30 years in the general population in Uganda

	Lifetime partners			Current regular partners			Inconsistent condom use^3^		
	
Psychological distress^1^	Proportion with 4 or more lifetime partners (%)	Crude OR (95% CI)	Adjusted^2 ^ OR (95% CI)	Proportion with more than 1 current partner (%)	Crude OR (95% CI)	Adjusted^2 ^ OR (95% CI)	Proportion with inconsistent condom use (%)	Crude OR (95% CI)	Adjusted^2 ^ OR (95% CI)
**Men**									
1st (lowest)	16/77 (20.8)	1	1	10/78 (12.8)	1	1	44/63 (69.8)	1	1
2nd	32/85 (37.6)	2.30 (1.14-4.65)	2.78 (1.31-5.92)	10/85 (11.8)	0.91 (0.36-2.31)	0.98 (0.36-2.62)	47/71 (66.2)	0.85 (0.41-1.75)	0.61 (0.24-1.55)
3rd	38/82 (46.3)	3.29 (1.63-6.64)	4.49 (2.09-9.66)	16/80 (20.0)	1.70 (0.72-4.02)	2.05 (0.81-5.19)	48/72 (66.7)	0.86 (0.42-1.79)	0.79 (0.31-2.06)
4th (highest)	37/86 (43.0)	2.88 (1.43-5.78)	3.58 (1.66-7.69)	13/86 (15.1)	1.21 (0.50-2.94)	1.20 (0.46-3.13)	65/75 (86.7)	2.81 (1.19-6.61)	3.42 (1.07-10.92)

**Women**									
1st (lowest)	2/61 (3.3)	1	1	1/61 (1.6)	1	1	27/40 (67.5)	1	1
2nd	18/87 (20.7)	7.70 (1.71-34.55)	8.47 (1.84-39.09)	1/87 (1.1)	0.70 (0.04-11.37)	0.62 (0.04-10.25)	54/70 (77.1)	1.63 (0.68-3.86)	2.13 (0.76-5.95)
3rd	16/81 (19.8)	7.26 (1.60-32.93)	8.13 (1.73-38.25)	4/80 (5.0)	3.16 (0.34-29.00)	4.59 (0.49-43.38)	52/65 (80.0)	1.93 (0.78-4.73)	2.62 (0.90-7.59)
4th (highest)	34/82 (41.5)	20.90 (4.78-91.44)	22.27 (4.89-101.35)	8/82 (9.8)	6.49 (0.79-53.32)	8.56 (1.00-73.19)	60/75 (80.0)	1.93 (0.81-4.60)	2.35 (0.82-6.76)

^1^ Numbers represent HSCL-25 quartiles.

^2 ^Adjustment for age, education, marital status and place of residence.

^3 ^Analyses pertain only to participants who had ever had sex.

Explorative adjustment of the above associations for alcohol use suggested that the associations between psychological distress and sexual risk behaviours were virtually independent of alcohol use in both men and women.

### Alcohol use and sexual risk behaviours

Alcohol use was independently associated with a greater number of lifetime sexual partners in both men and women, after simultaneous adjustment for socio-demographic variables, see Table 4. Moreover, alcohol use was independently associated with having concurrent sexual partners in both men and women. Associations between alcohol use and inconsistent condom use did not reach statistical significance in either men or women.

**Table 4:** Association of alcohol use with sexual risk behaviours in 334 men and 312 women aged 18-30 years in the general population in Uganda

	Lifetime partners			Current regular partners			Inconsistent condom use^2^		
	
Alcohol use	Proportion with 4 or more lifetime partners (%)	Crude OR (95% CI)	Adjusted^1 ^ OR (95% CI)	Proportion with more than 1 current partner (%)	Crude OR (95% CI)	Adjusted^1 ^ OR (95% CI)	Proportion with inconsistent condom use (%)	Crude OR (95% CI)	Adjusted^1 ^ OR (95% CI)
**Men**									
Less than once per week	64/213 (30.0)	1	1	20/211 (9.5)	1	1	108/166 (65.1)	1	1
At least once per week	59/116 (50.9)	2.41 (1.51-3.85)	1.82 (1.09-3.03)	29/117 (24.8)	3.15 (1.69-5.87)	2.11 (1.08-4.11)	95/114 (83.3)	2.69 (1.49-4.83)	1.70 (0.83-3.48)

**Women**									
Less than once per week	45/268 (16.8)	1	1	6/268 (2.2)	1	1	159/208 (76.4)	1	1
At least once per week	25/41 (61.0)	7.74 (3.83-15.66)	7.75 (3.63-16.57)	8/41 (19.5)	10.59 (3.46-32.40)	11.88 (3.66-38.53)	34/40 (85.0)	1.75 (0.69-4.40)	1.99 (0.68-5.79)

^1 ^Adjustment for age, education, marital status and place of residence.

^2 ^Analyses pertain only to participants who had ever had sex.

When the above associations were adjusted for depression and psychological distress, respectively, the associations between alcohol use and sexual risk behaviours remained virtually unchanged in both men and women.

## Discussion

### Main findings

Depression, psychological distress and alcohol use were all associated with having a greater number of lifetime sexual partners and with having concurrent sexual partners, with stronger associations found among women. Moreover, although less consistent, associations were found between mental health indicators and inconsistent condom use, with stronger associations found among men.

All the above associations were adjusted for confounding by socio-demographic variables. Explorative analyses suggested that the associations of depression and psychological distress with sexual risk behaviours were virtually independent of alcohol use. Conversely, the association between alcohol use and sexual risk behaviours was virtually independent of depression and psychological distress.

The prevalence of depression was in line with earlier Ugandan studies[23,24] but lower than the extremely high prevalence recently reported in war-torn Northern Uganda[25,26]. Heavy episodic drinking at least once per week was common, consistent with reports suggesting that Uganda has one of the world's highest alcohol per capita consumptions[38]. The mean number of lifetime partners among persons who had ever had sex was broadly in the same range as that reported in a national survey conducted at the time of this study[22]. The point prevalence of concurrency was relatively low, although consistent with previous Ugandan studies on concurrency[39,40]. Inconsistent condom use was reported by approximately 75% of those who had ever had sex, in agreement with reports of low rates of condom use at last sexual intercourse from the period when this study was performed[22].

### Interpretation

Depression was associated with past and current multiple partners in women. A number of interpretations for these associations are possible. Firstly, depression and multiple partners may be indirectly linked through a common cause. For instance, poverty with food insecurity may cause poor mental health, and may also force women into transactional sexual relationships in order to obtain food[41]. Similarly, having an abusive partner may lead to depression, while also motivating women to look for extramarital or alternative partners. Secondly, depression might contribute to having multiple partners. Thus, fatalism and hopelessness may lead to disregard of the threat of HIV and thus hypothetically contribute to multiple sexual partnerships[42]. Moreover, depressed women may be more vulnerable to being coerced into sex, potentially leading to an increased number of sexual contacts[20]. Thirdly, having multiple partners might lead to depression. Thus, having multiple partners may cause HIV related worries[43]. Moreover, STD and HIV infection increase the risk of subsequent depression[4,10]. In addition, women with multiple partners in Uganda may have feelings of guilt and may fear being labelled as promiscuous[44]. More research is needed in order to clarify the meaning of the association between depression and multiple partners in the Ugandan context.

Similarly, the associations between psychological distress and past and current partners were stronger in women. Studies in high-income countries suggest that women having multiple partners may have particularly poor mental health[11,12]. Psychological distress and depression partly overlap, and factors contributing to multiple partners in depressed women could also be operating with regard to psychological distress. In addition, psychological distress may lead to casual sex as a coping strategy, and may also decrease self-efficacy for changing risky habits[45]. Lack of sense of the future may lead to non-planning and impulsivity[46]. In addition, psychological distress may cause destructiveness[29], potentially contributing to sexual risk behaviours.

In contrast, the associations of depression and psychological distress with inconsistent condom use were stronger among men. Men's mental state may have a greater influence than that of women on the condom-use decision, given that men generally decide over condom use in Uganda[47].

While associations between alcohol use and sexual risk behaviours have previously been found in Uganda[48], the mechanisms explaining the association between alcohol use and sexual risk behaviours in this setting, or elsewhere, are not fully understood: Clearly, alcohol consumption causes behavioural disinhibition potentially leading to risky decisions. However, alcohol use may also be a marker for a psychological trait, or state, conducive to risky sexual behaviours[49,50], e.g. depression or psychological distress. In the context of this uncertainty, the current findings, although cross-sectional, do suggest that alcohol use and depression/psychological distress mainly influence sexual risk behaviours through mutually independent causal pathways.

In summary, the interrelationship between poor mental health and sexual risk behaviours in the Ugandan setting is likely complex, and may well be bi-directional.

### Methodological considerations

The current study was cross-sectional and its aim was to assess association. Thus, the interpretations presented regarding the possible nature and direction of the associations should be viewed as hypotheses for further testing.

Our measure of concurrency was crude, although standard definitions of concurrency are still under development[51]. The measure involved some portion of subjectivity given that what is perceived as a current regular sexual partner depends on expectations of future encounters. However, 'current' regular relationships have been assessed also in previous studies[35,39,40]. Moreover, the measure was not subject to recall bias since participants were not required to reconstruct past partnerships. In addition, recall and concentration is affected by depressive symptoms[52] and an assessment of dates and frequencies of sexual behaviours in the past would potentially have entailed significant risks of bias.

As in much survey research on sensitive topics, responses may be subject to social desirability bias. Having multiple sexual partners is often not socially desirable for women in Uganda, and the numbers of partners and rates of concurrency reported among women should be viewed as minimum estimates. However, the rates found are largely consistent with evidence from previous studies on concurrency from Uganda[39,40].

The sampling method used was not random, since villages/neighbourhoods were purposively selected. Thus, the socio-demographic profile of the sample does not necessarily reflect that of the districts where the study was performed. In the current sample, the proportion of persons having completed primary school was higher and the proportion of persons who were married or cohabiting was lower than in the general population in Uganda [53], although it should be noted that only persons aged 18-30 years were included in the study. While the prevalence estimates should thus be interpreted with some caution, the purposive sampling of villages/neighbourhoods representing a broad range of economic development arguably increases the extent to which the associations found may be applicable to external populations from different socio-economic strata.

Follow-up visits were not performed for households where no one was at home at the time of the interviewer's visit. If persons who were not at home systematically differed from those at home with respect to mental health and sexual risk behaviours, a selection bias could hypothetically have been introduced into the study.

No measure of personal or household wealth was included. However, results were adjusted for a proxy measure of socio-economic status (i.e. education) and place of residence.

The alcohol measure was crude and did not capture nuances in alcohol consumption, such as the exact number of drinks per heavy episodic drinking occasion. However, when investigating the behavioural impact of heavy episodic drinking, solely assessing the number of standard drinks may have limitations, given that the number of drinks required for altering behaviour is individual[54]. Thus, combining self-reports of heavy episodic drinking with standard drink counts may provide the best option for research on alcohol and sexual risk behaviours, particularly in settings where the 'standard drink' concept is difficult to operationalise.

## Conclusions

To our knowledge, this is the first population-based study to demonstrate an association of depression and psychological distress with sexual risk behaviours in a low-income sub-Saharan African setting. Although preliminary, the current findings indicate that the association between poor mental health and sexual risk behaviours may be present across both high and low-income settings, despite radical contextual differences e.g. in terms of economic wealth, gender inequality, level of urbanisation, personal mobility and religious norms, i.e. structural factors of relevance for sexual risk behaviours[14,18].

Indeed, the current findings are consistent with the notion that depression, psychological distress and alcohol use are risk factors for sexual risk behaviours also in sub-Saharan African low-income settings, although longitudinal studies are needed in order to confirm this. Depression, psychological distress and alcohol use are prevalent in many countries with generalised HIV epidemics[6,23-26,55,56]. Thus, assuming causality, these conditions could potentially have considerable impact on population rates of sexual risk behaviours in low-income countries with high HIV prevalence.

Improving mental health may theoretically decrease sexual risk behaviours. Based on our findings and the evidence from high and middle-income countries[9-12,19-21,45] we support the call for mental health intervention trials to include sexual risk behaviour and biological variables as outcome measures[2], particularly in low-income settings with generalised HIV epidemics. Moreover, qualitative studies should further explore subjective experiences of how poor mental health and sexual risk behaviours are inter-connected in low-income settings[57].

Irrespective of the direction of causality, the mere co-existence of poor mental health and sexual risk behaviours has implications for HIV prevention: Those with the greatest HIV prevention needs may not always be sufficiently psychologically fit to benefit from intervention messages. For instance, learning to be assertive when communicating about sex with one's partner may be difficult for a woman who is depressed and anxious. HIV intervention trials should assess to what extent participants' mental health at baseline influence intervention outcomes. Moreover, HIV preventive programmes may need to consider including mental health and alcohol use reduction components into their intervention packages, in settings and groups where depression, psychological distress and alcohol use are common.

## Competing interests

The authors declare that they have no competing interests.

## Authors' contributions

PL designed and coordinated the study, performed analyses and wrote the manuscript. GR and SA led the data collection. AT and PA contributed to data analysis and results interpretation. PO contributed to study design, data analysis and results interpretation. EC conceived of the study and contributed to all stages of the work. All authors read and approved the final manuscript.

## Pre-publication history

The pre-publication history for this paper can be accessed here:

http://www.biomedcentral.com/1471-2458/11/125/prepub
